# Effect of Back Plate Preheating Assistance System and Deep Rolling Process on Microstructure Defects and Axial Force Reduction of Friction Stir Welded AA6061 Joint

**DOI:** 10.3390/ma17184447

**Published:** 2024-09-10

**Authors:** Pinmanee Insua, Wasawat Nakkiew, Adirek Baisukhan, Siwasit Pitjamit

**Affiliations:** 1 Department of Industrial Engineering, Faculty of Engineering, Chiang Mai University, Chiang Mai 50200, Thailand; pmn.insua@gmail.com (P.I.); adirek.b@cmu.ac.th (A.B.); 2 Department of Industrial Engineering, Faculty of Engineering, Rajamangala University of Technology Lanna, Chiang Mai 50300, Thailand; siwasit.pitjamit@rmutl.ac.th

**Keywords:** friction stir welding, preheating assistance system, deep rolling, axial force, temperature variation, residual stress, industry innovation and infrastructure

## Abstract

This study investigates the effects of a back plate preheating assistance system and deep rolling (DR) on axial force and tunnel defects during friction stir welding (FSW). Different preheating configurations—advancing side (AS), retreating side (RS), and both sides—were examined to evaluate their impact on axial force reduction, temperature distribution, and defect minimization. Axial force measurements were taken using a dynamometer, and temperature histories were recorded with a thermal camera. The results demonstrate that a preheating temperature of 200 °C is optimal, reducing axial force by 30.24% and enhancing material flow. This temperature also facilitated deeper tool penetration, especially when preheating was applied to both sides. Preheating on the AS resulted in the smallest tunnel defects, reducing defect size by 80.15% on the RS and 96.91% on the AS compared to the non-preheated condition. While DR further reduced tunnel defects, its effectiveness was limited by the proximity of defects to the surface. These findings offer significant insights for improving the FSW process.

## 1. Introduction

Friction Stir Welding (FSW) represents one of the advanced joining processes highly regarded for its applicability to materials that are challenging to weld, such as aluminum alloys (AA). This is particularly pertinent to alloys in the AA2xxx, AA6xxx, and AA7xxx series, which are characterized by their low melting temperatures. FSW is a non-consumable technique that employs a rotational pin, devoid of an external heat source, while the material remains in a solid state [1]. The efficacy and superior quality of weldments produced via FSW have been extensively validated by numerous researchers. Prior studies have predominantly concentrated on optimizing process parameters tailored to specific materials, including welding speed, rotational speed, pin shape, and plunge depth. For instance, Trimble et al. [2] demonstrated that a threaded cylindrical pin exhibits lower susceptibility to damage in AA2024-T3 joints compared to a smooth cylindrical pin, as the threaded design promotes greater material deformation. Similarly, Motalleb-Nejad et al. [3] explored the influence of pin profiles on the microstructure and mechanical properties of magnesium alloys, concluding that tapered and screw-threaded cylindrical pins generate defect-free joints more effectively than other pin profiles. Consequently, threaded pin designs have been identified as providing superior performance relative to their counterparts. Nevertheless, parameter optimization within the Friction Stir Welding (FSW) process alone may not suffice to achieve optimal weld properties. This recognition has spurred a plethora of subsequent investigations into various post-weld enhancement methods such as post-weld heat treatment, shot peening, coating, and the deep rolling process. On the other hand, some assistive techniques can help improve material properties during the FSW process, regardless of adjusting the process parameters. Scholars have revealed different techniques to apply along with the welding setup. The work of Verma et al. [4] studied the effects of preheating and water cooling during the FSW process. They found that preheating not only improves the ductility of the joint but also provides fine grain in the nugget zone.

Numerical simulations and experimental endeavors in the realm of preheating assistance have witnessed a surge, fueled by a collective pursuit of unraveling the intricate thermal dynamics governing subsequent welding processes. Various types of heat sources have been applied, such as back hot plates [4,5,6,7], laser [8,9,10], plasma [11,12,13], induction coil [14], heat gun [15], and nozzle [16]. Scholars such as Ji et al. [5] and Kim et al. [7] have delved into the temperature-dependent mechanical behaviors of various materials, providing valuable insights into the complex relationship between preheating and resultant weld quality. Wada et al. [10] found that laser preheating was able to reduce defect formation and tool rotational torque during FSW. Additionally, plasma preheating ahead of the FSW tool significantly reduced grain size and vertical force [13]. Previous studies have investigated the effects of plate preheating on the microstructural evolution during FSW, highlighting the sensitivity of material properties to temperature variations. These studies underscore the pivotal role of preheating in influencing the overall quality and performance of welded joints, primarily applied to Inconel and steels. However, the influence of preheating sides, particularly on aluminum alloy plates, which are challenging to weld, has not been extensively addressed. Therefore, our examination of preheating conditions aligns with an evolving body of literature emphasizing the importance of temperature control in achieving optimal material outcomes. Furthermore, we aim to simplify and reduce costs using techniques such as back plate preheating. Deep rolling (DR) is a grain refinement process. Upon application to the specimen surface, deep rolling induces plastic deformation through external force, causing dislocations to accumulate or entangle within the coarse grains [17,18]. These dislocations subsequently rearrange into sub-grain boundaries, a crucial step for the formation of fine grains and compressive stress, as large tensile residual stresses can be produced during welding processes [19]. Insights from previous works [20,21,22] emphasize the critical role of deep rolling in refining microstructures and enhancing fatigue performance in welded components. Additionally, DR can avoid tunnel defects caused by insufficient material flow in FSW due to the applied pressure.

**Figure 1:**
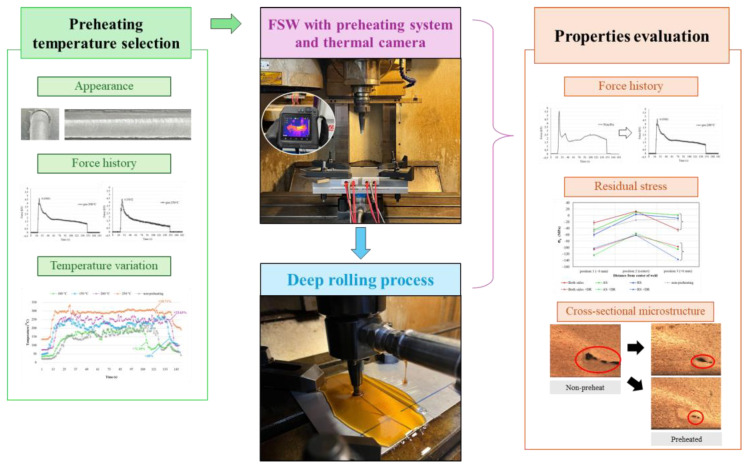
Schematic flowchart illustrating the methodology of this study.

Tunnel defects are among the most critical issues in the FSW process. These defects typically arise due to insufficient material flow during welding, leading to the formation of irregular welds [23,24]. Tunnel defects occur when the material flow around the tool pin is inadequate, resulting in an incomplete weld fill [25,26]. This defect significantly compromises mechanical properties, as larger tunnel sizes correlate with greater degradation [27]. Rasti [28] observed that in the FSW of 1060 commercially pure aluminum, a minimum heat input of approximately 800 J/mm is necessary to mitigate tunnel voids. Furthermore, tunnel defects have a pronounced impact on the mechanical properties of welded joints. For instance, Balos and Sidjanin [29] reported that the ultimate tensile strength (UTS) efficiency of joints with tunnel defects was reduced by 25–82% compared to defect-free joints. Therefore, achieving optimal temperature and material flow during FSW is crucial to preventing tunnel defects and maintaining the mechanical integrity of the weld. Hence, this study aims to improve weldment of the FSW process by applying the back plate preheating assistance system as well as the deep rolling process, which reduces axial force and promotes the material flow. Our research focuses on a 150 mm × 200 mm × 3 mm aluminum alloy plate with a preliminary preheating temperature range of 100 °C to 250 °C. Utilizing numerical analysis, we seek to contribute to the discourse on optimal preheating conditions by determining the temperature at which the aluminum alloy plate exhibits improved formability and structural performance during subsequent FSW and deep rolling processes. The workflow is depicted step by step in Figure 1. Additionally, this study aims to deepen the understanding of the relationship among temperature, axial force, and residual stress, advancing the state of knowledge in the pursuit of precision and reliability in advanced welding techniques. 

## 2. Materials and Methods

### 2.1. Workpiece Materials

In this study, 3 mm thick 6061 aluminum alloy plates were used for the experiment. The dimensions of the plate are 150 mm × 100 mm × 3 mm. The chemical composition measured using the energy-dispersive X-ray fluorescence (EDXRF) method with the JEOL 105 model JSX3400R (JEOL manufacturer, Tokyo, Japan) is shown in Table 1.

### 2.2. Friction Stir Welding (FSW)

**Figure 2:**
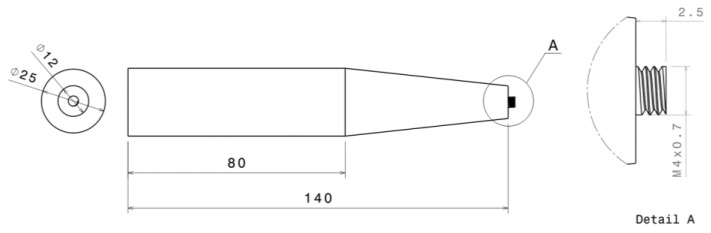
A schematic dimension and geometry of the cylindrical threaded tool [31]. Scale is in mm.

**Figure 3:**
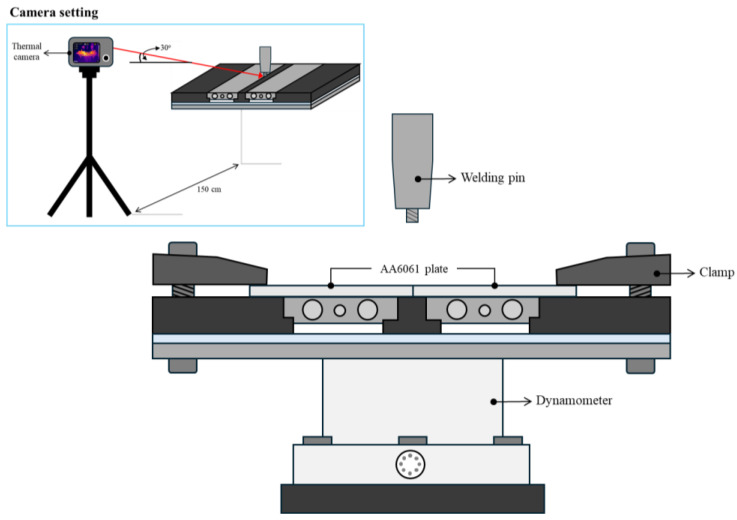
The support system and welding setup.

The FSW process was conducted using CNC model VMC500 (Bridgeport, UK) in collaboration with a force measurement device called a dynamometer and an infrared camera. High carbon steel SKD61 was milled into a cylindrical threaded tool designed by setting a pitch of 0.7 mm and major diameter of 4 mm, as shown in Figure 2. The tool’s mechanical properties were improved by heat treatment, starting with hardening at 1035 °C, followed by tempering at 520 °C for 3 times in the vacuum furnace, and quenching in N_2_ to achieve a hardness of 56 HRC. The welding parameters were set to a rotational speed of 980 rpm, a welding speed of 65 mm/min, and a plunge depth of 2.75 mm [30]. The dynamometer was utilized to measure axial force (z-force), torque, and x-force during FSW. Accurate force measurements provide essential data for evaluating the effects of the back plate preheating assistance system on the welding process. The methodology emphasizes the importance of precise force control for optimizing FSW outcomes. In this work, the dynamometer was positioned under the back plate preheating assistance system. Relevant force variations were simultaneously generated using DynoWare software (version 3.2.2.0, KISTLER manufacturer, Winterthur, Switzerland). The force data was collected at a frequency of once per second, with a time range of 180 s for each welding. To collect temperature data, an infrared camera, the FLIR model T560 (Teledyne FLIR, Wilsonville, OR, USA), was employed to monitor temperature variations during FSW and thermal images. The camera has a resolution of 640 × 480 pixels, an object temperature range of 0 °C to 650 °C, and a frame rate of 30 Hz. This non-intrusive approach allows real-time temperature measurements, offering insights into the thermal dynamics influenced by the back plate preheating assistance system. The welding setup and welding fixed parameters are shown in Figure 3 and Table 2, respectively.

### 2.3. Preheating Process

**Figure 4:**
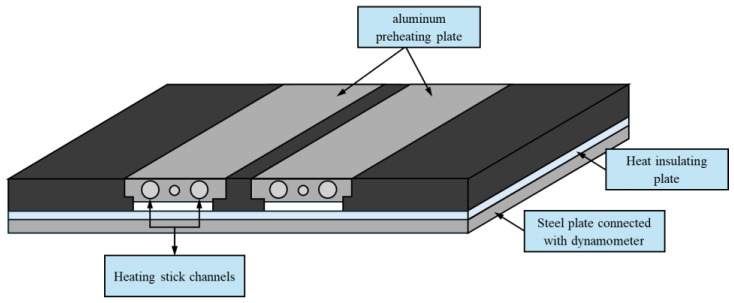
The back plate preheating assistance system.

A back plate preheating assistance system was built to deliver heat into the workpiece during the welding process. The components of the back plate preheating assistance system are explained in Figure 4. Prior to the preheating process, a preliminary experiment was conducted to determine the appropriate temperature parameters for the remainder of this study. The aluminum alloy plate was welded with the back plate preheating assistance system, which varies temperatures of 100 °C, 150 °C, 200 °C, and 250 °C. This preliminary temperature range aims to encompass a spectrum of thermal conditions for subsequent investigations. Firstly, two AA6061 plates were placed on the back plate. Afterward, the heat source was turned on. Once the temperature increased from room temperature to the desired level, a 7-min countdown was initiated before commencing the welding process. Each welding condition was maintained for a duration of 2.5 min.

**Figure 5:**
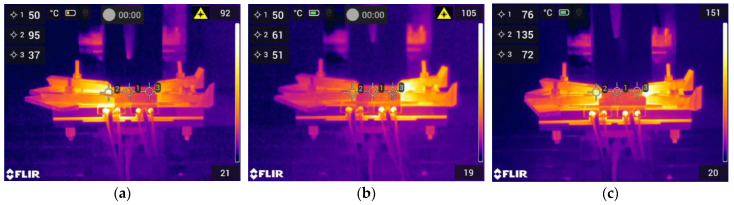
Thermal images of (**a**) preheating on the retreating side, (**b**) preheating on the advancing side, and (**c**) preheating on both sides.

Since a proper temperature was found. Four preheating conditions were set at the same temperature. This preheating experiment is intended to isolate and analyze the impact of preheating on specific aspects of the weldment. Non-preheating serves as a baseline, while preheating on the advancing side, retreating side, and both sides allow for a nuanced exploration. Figure 5 presents various thermal camera images captured during preheating on different sides. All steps of the preheating process were performed as explained in the preliminary experiment.

### 2.4. Deep Rolling Process (DR)

**Figure 6:**
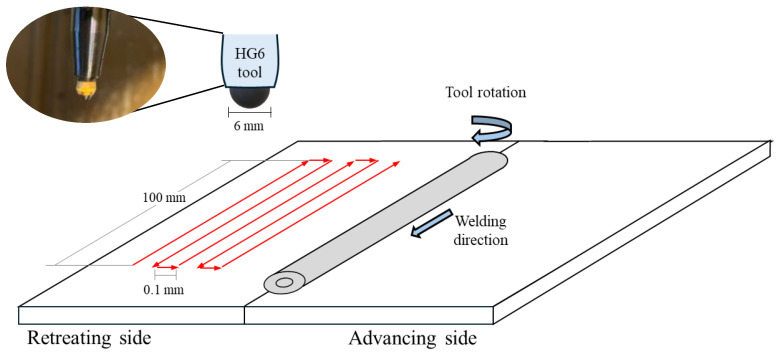
The support system and deep rolling setup. (The red arrow indicates the direction of deep rolling).

The DR process was implemented to enhance the surface mechanical properties of welded joints. DR is crucial for understanding the synergistic effects of preheating on subsequent mechanical treatments. In this instrumentation, HG6 (ECOROLL, Milford, OH, USA) was applied. The HG6 ball, with a diameter of 6 mm, was pressed onto the surface of the welded workpieces and moved with a feed rate of 1400 rpm. A high-pressure hydraulic pump provided an external pressure supply of 150 bar to the hydrostatic tool. Hydraulic oil, specifically TOTAL model Lactuca LT3000, was used without any mixing. The oil was delivered to the DR system by an immersion pump with a flow rate of 2 L per minute during the DR process. The hydrostatic tool was installed in the CNC machine (Bridgeport, UK). The DR area was designed to be 70 mm wide by 100 mm long. The parameters in the DR process are shown in Table 3. Figure 6 shows DR direction in both longitudinal and transverse directions. The tool began pressing from the lower left corner and ended at the upper right corner. Table 4 presents the preheating and deep rolling conditions utilized in this investigation.

### 2.5. Mechanical Properties

**Figure 7:**
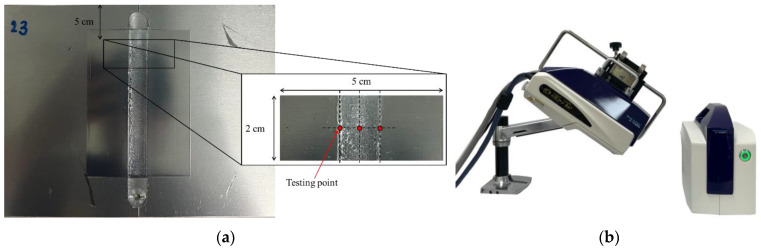
(**a**) Part of workpiece evaluated residual stress. (**b**) Residual stress analyzer.

Residual stress and microhardness assessments were performed to evaluate the material properties post-welding, with the inclusion of preheating assistance and deep rolling. These measurements provide a comprehensive analysis of the impact of plate preheating on structural integrity and mechanical performance. Liu et al. [32] have contributed methodologies and insights pertinent to the evaluation of residual stress and microhardness in welded components. Residual stress was quantified through X-ray diffraction (XRD) employing the cos-α technique. The cos-α method, initially proposed by Taira, Tanaka, and Yamasaki in 1978 [33], is utilized for in-plane biaxial stress analysis. This method leverages the Debye ring obtained from a single measurement using a two-dimensional detector [34]. Each specimen, as depicted in Figure 7a, was excised from the welded zone for testing. Figure 7b illustrates the residual stress analyzer (PULSTEC manufacturer, Hamamatsu, Shizuoka, Japan) model µ-x360, utilized in this study. X-ray incidence angle, irradiation time, and level of ambient light are 35.0 deg, 90 s, and 0.3%, respectively.

## 3. Results and Discussion

### 3.1. Results from Preheating Temperature Selection

**Figure 8:**
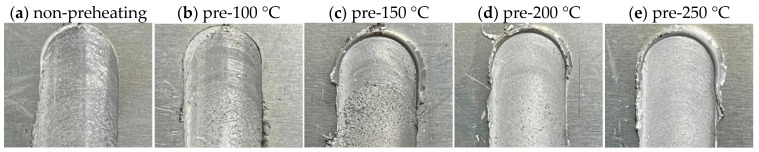
Surface appearance of plunging area under different preheating temperatures: (**a**) non-preheating, (**b**) preheated at 100 °C, (**c**) preheated at 150 °C, (**d**) preheated at 200 °C, and (**e**) preheated at 250 °C.

**Figure 9:**
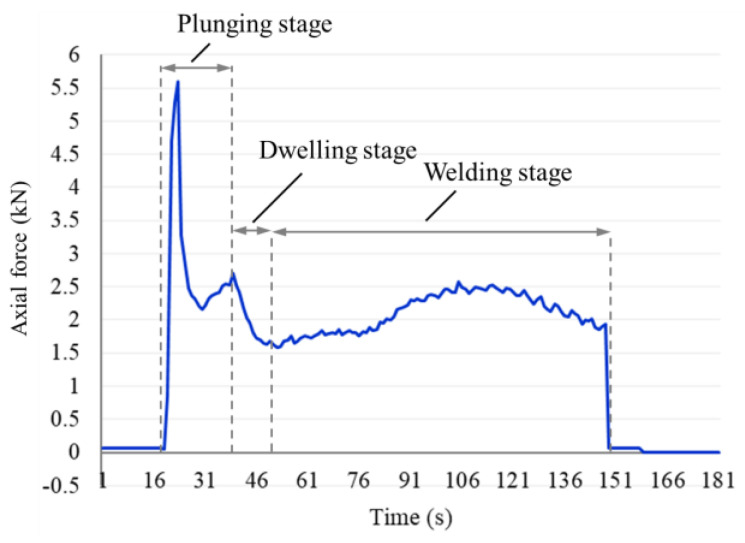
Stages of axial force distribution during the FSW process (non-preheating).

Figure 8 shows the initial areas of the weldment obtained through the plunging and dwelling steps, which are important phases controlling the entire welding performance and involving forces [35]. This phase occurs after the tool has penetrated the metal and rotated for 3 s. Each preheating temperature evidently affects the areas, as illustrated in the differences in Figure 9. Higher temperatures create more flash around the initial area since preheating provides an additional heat source that causes early, quick, and high degrees of deformation and easy flow of plasticized material around the tool contact surface [36]. Non-preheating and 100 °C preheating conditions produce less flash compared to others. On the other hand, at 250 °C, the aluminum alloy metal is excessively molten not only around the initial area but also along the length until the end of welding, affecting the surface appearance quality, as shown in Table 5. Therefore, for a specific material, the higher limits for the preheating temperature should be taken into account to create defect-free welds.

**Figure 10:**
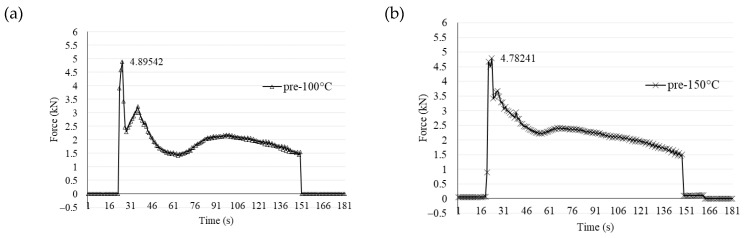

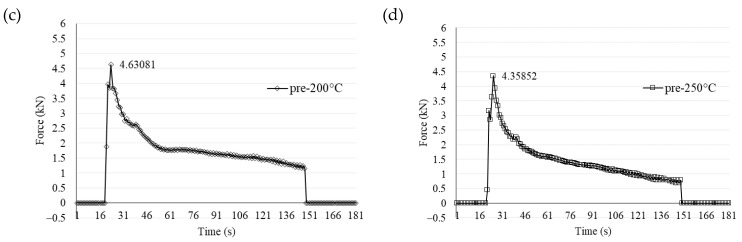
Axial force variations under different preheating temperatures: (**a**) preheated at 100 °C, (**b**) preheated at 150 °C, (**c**) preheated at 200 °C, and (**d**) preheated at 250 °C.

Axial force immensely impacts the FSW process [37]. Previous studies [38,39] have shown that an axial force distribution in the FSW process can be divided into three stages, including plunging, dwelling, and welding. The profile depends on tool movement and material flow during the process. Figure 10 shows an axial force distribution of the non-preheating condition. It found that the peak axial force appears in the plunging stage as the rotating pin initially penetrates the metal. Then, in the dwelling stage, the tool remains rotating for 15 s to generate frictional heat before moving forward. Finally, in the welding stage, the pin moves straight for 150 mm.

Preheating the aluminum alloy plates on both the advancing and retreating sides at various temperatures during the welding process resulted in a gradual decrease in axial force histories with increasing temperatures, as illustrated in Figure 10. The maximum axial forces exhibited a similar trend, decreasing with higher preheating temperatures. Specifically, the maximum axial force for non-preheating plates was measured at 5.60577 kN, while the maximum axial forces at preheating temperatures of 100 °C, 150 °C, 200 °C, and 250 °C were 4.89542 kN, 4.78241 kN, 4.63081 kN, and 4.35852 kN, respectively. This reduction in axial force can be attributed to the effect of preheating, which decreases the plunging force required during the friction stir welding (FSW) process [40]. Furthermore, the curve became smoother with higher preheating temperatures, as high-temperature preheating reduces the axial force required during the welding process.

**Figure 11:**
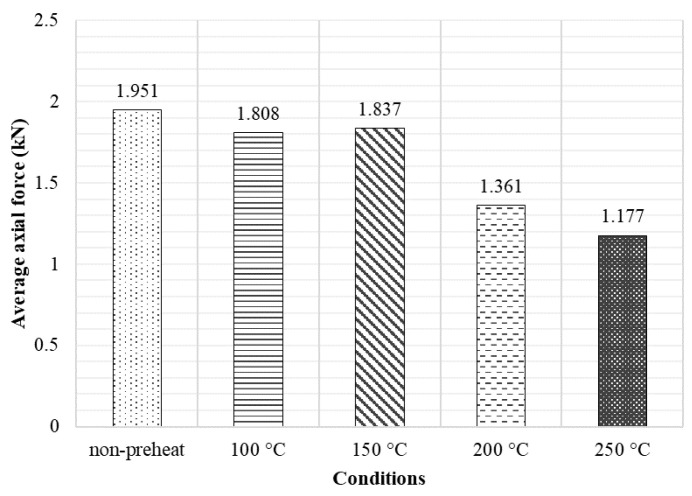
Average axial force in the welding stage.

Figure 11 presents the average axial force observed during the welding stage at various preheating temperatures. This data elucidates the reduction in axial force in a crucial area, as the welding stage represents the longest segment of the welding process. The average axial force decreases along with the higher preheating temperature. The average axial force during the welding stage, when preheated at 100 °C, 150 °C, 200 °C, and 250 °C, decreased from the non-preheating condition by 7.33%, 5.84%, 30.24%, and 39.67%, respectively. It demonstrates that putting heat into the metal sheet before beginning the welding process highly avoids overload force in axis Z.

**Figure 12:**
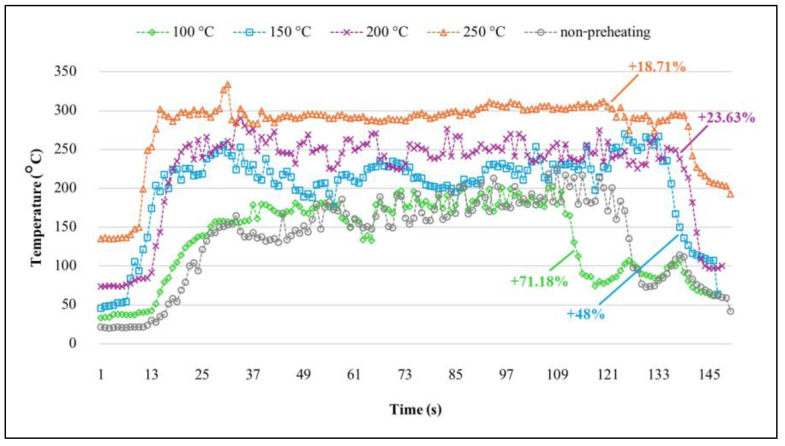
Temperature distribution at different preheating temperatures.

Figure 12 illustrates the real-time temperature variations observed during the welding process under different preheating conditions. The data demonstrate that temperature trends increase proportionally with higher preheating temperatures. Initially, the recorded temperatures were below the preheating temperature; however, they began to rise following the tool’s penetration into the metal and eventually stabilized. Notably, the temperatures at 200 °C and 250 °C exhibited the greatest stability among all conditions. The average temperatures during the welding stage, when preheated at 100 °C, 150 °C, 200 °C, and 250 °C, with a processing time of approximately 92 s, increased from the initial preheating temperature by 71.18%, 48%, 23.63%, and 18.71%, respectively, as shown by the colored lines in Figure 12. Consequently, these preliminary results suggest that a preheating temperature of 200 °C is optimal, considering the resulting appearance, force requirements, and temperatures achieved. This temperature will be employed in the subsequent phase of this study.

### 3.2. Influence of Preheating Positions on Thermal and Force Variations

**Figure 13:**
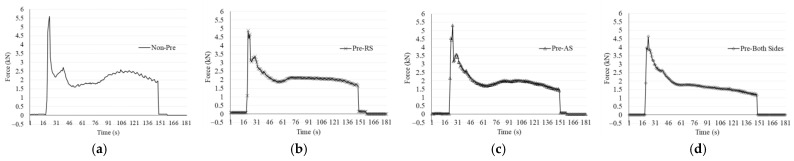
Axial force variations under different preheating conditions: (**a**) non-preheating, (**b**) preheating on retreating side, (**c**) preheating on advancing side, and (**d**) preheating on both sides.

Preheating positions provide different profiles of axial force, as illustrated in Figure 13. It was observed that the highest frustrated variation occurred in the non-preheating condition, as shown in Figure 13a. While preheating on the retreating side in Figure 13b has the most stable force history in the welding stage compared to others. Preheating on both sides achieved the least axial force for all stages. This phenomenon was affected by a high temperature distribution inside materials. The material was softened wider by preheating assistance, causing high material flow [16]. When the pin rotated, there is a resistant force between the pin and metal, yet the preheating assistance system helps decrease stiffness and unsmoothness. As reported by Garg et al. [41] that preheating during FSW of dissimilar AA6061-AA7075 significantly reduced welding forces. Thus, the smooth profiles in the welding stage in Figure 13b–d were obtained compared to non-preheating. Which implies that tool wear.

### 3.3. Residual Stress Distributions

**Figure 14:**
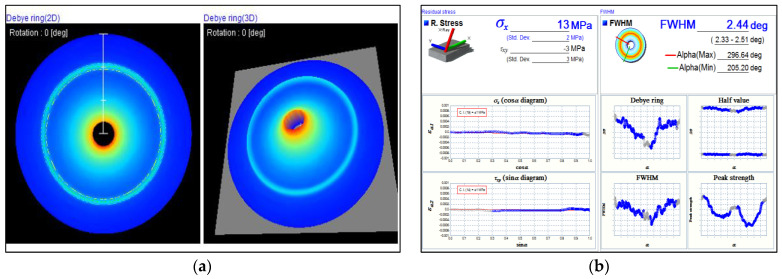
(**a**) Debye rings in 2D and 3D from residual stress testing under the condition of preheating on both sides without deep rolling; (**b**) Display of residual stress values calculated using the cos α technique. This condition exhibits the highest positive value (tensile stress).

**Figure 15:**
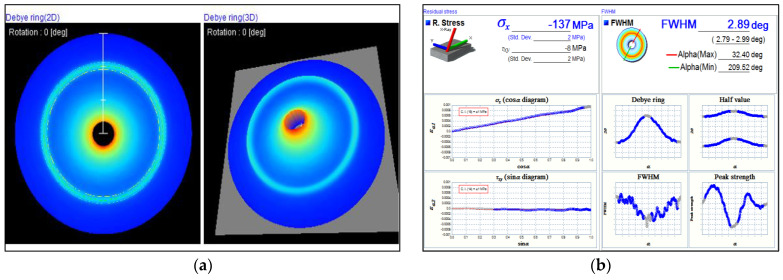
(**a**) Debye rings in both 2D and 3D from residual stress testing under the condition of preheating on the retreating side with deep rolling; (**b**) Display of residual stress values calculated using the cos α technique. This condition exhibits the highest compressive stress compared to all other conditions.

For the evaluation of residual stress, Debye rings were generated using the cos α (alpha) technique applied to the specimen surface. Each testing point produced a distinct Debye ring, visible in both 2D and 3D, as illustrated in Figure 14a. The residual stress values calculated by the cos α technique, along with various diagrams corresponding to the Debye rings, are presented in Figure 14b. Figure 14 shows a Debye ring representing the highest positive value or tensile stress observed in this study. In contrast, Figure 15 displays the Debye ring associated with the highest compressive stress. The differences between tensile and compressive stresses can be observed by comparing the Debye rings and the full width at half maximum (FWHM) profiles in Figure 14b and Figure 15b.

**Figure 16:**
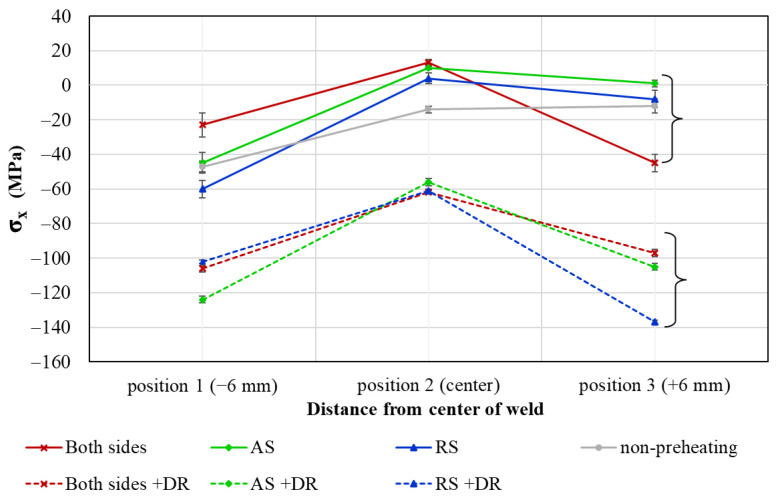
Residual stress at different positions on specimen surface.

The residual stress measurements obtained from positions 1 to 3 under various conditions are plotted in one diagram (Figure 16). Error bars were added individually for each data point. The same preheating sides are indicated by the same color lines, with different conditions for deep rolling (DR) represented by dashed lines. It was observed that all specimens subjected to DR exhibited compressive residual stress, while specimens without DR showed partial tension stress. The center of the welds (position 2) exhibited higher tensile stress compared to positions 1 and 3, which are located at the tool shoulder edge. Compared to the as-welded specimens, preheating without DR shifted the residual stress towards more positive values, as seen in Figure 16. The red, green, and blue lines are plotted above the grey line (as-welded). All dashed lines associated with DR are in the compressive region. The influence of DR decreased the residual stress to negative values, promoting the formation of fine grains. After undergoing DR, the differences in residual stress among the three positions were reduced.

Table 6 presents the percentage difference in residual stress between non-deep rolled and deep rolled specimens. The smallest percentage difference, 70.0%, is observed at position 1 under the preheating RS condition. In contrast, a significant percentage difference in residual stress was noted at the right shoulder under the preheated AS condition, where the residual stress changed from 1 MPa to −105 MPa after applying deep rolling pressure. The most compressive residual stress of −137 MPa was observed at the right shoulder under preheating on the AS. Additionally, deep rolling on the preheating AS condition mitigates the disparity in residual stress between positions 1 and 3, which correspond to the left and right tool shoulders, respectively. This observation is consistent with the findings of Salih et al. [42] and Schubnell et al. [43]. However, when evaluated from the surface, the effect of preheating assistance on residual stress is negligible.

### 3.4. Microstructure Analysis

To illustrate the effects of preheating on material flow behavior, the joint cross-sectional microstructures are detailed in Table 7. 

The microstructure of the stir zone (SZ) was analyzed using an optical microscope at 50× magnification. Table 7 illustrates the substantial impact of preheating and deep rolling on tunnel defects within the weld zone. Under non-preheated conditions, large tunnel defects were observed, with the largest defect on the retreating side (RS) measuring 0.0325 mm² and the most significant defect on the advancing side (AS) measuring 0.0551 mm². These defects are attributed to insufficient heat input and inadequate plasticized metal flow and mixing, consistent with the findings of Aldanondo et al. [46]. Similarly, Wada et al. [10] reported the occurrence of multiple tunnel defects within the nugget zone, primarily due to the absence of laser preheating. The force measurements in this study indicate that the axial force required under all preheating conditions was notably lower than that required for non-preheated conditions. Furthermore, the distance between the bottom surface and the tool end was narrower (the tool went deeper) under preheated conditions, suggesting an increased tool plunge depth into the metal. The plunge depth and groove depth are critical factors influencing the joint’s tensile properties [47], as highlighted by Rathee et al. [48], who observed that low plunge depths result in reduced material flow and cavity formation at the center of the SZ due to diminished heat generation and limited contact between the tool shoulder and base metal.

When a preheating plate assistance system was employed in three configurations—preheating on the RS, the AS, and both sides—the results in Table 7 demonstrated the following effects on tunnel defects: (i) Preheating on the RS reduced tunnel defect size by approximately 51.38% on the RS and 49% on the AS. (ii) Preheating on the AS reduced tunnel defect size by 80.15% on the RS and 96.91% on the AS. (iii) Preheating on both sides reduced tunnel defects by 91.65% on the RS and by 88.92% on the AS. These findings suggest that preheating during friction stir welding significantly contributes to the development of joints free from tunnel defects [40]. Preheating on the AS, in particular, enhances material flow from the retreating to the advancing side of the weld, as observed by Ajri et al. [49]. Inadequate stirring of the material on the AS occurs due to insufficient material softening and low plastic flow, as the temperature at the tool pin tip remains below 450 °C while the tool shoulder region exceeds 450 °C. Thus, increasing the temperature beneath the specimen can soften the metal prior to stirring. However, preheating alone is not the sole factor in mitigating defects.

Preheating followed by deep rolling (DR) further reduced tunnel defect sizes across all conditions. As shown in Table 5, the impact of DR on microstructure and tunnel defect size reduction is as follows: (i) Preheating on the RS followed by DR reduced tunnel size by 95.69% on the RS and 96.73% on the AS. (ii) DR post-preheating on the AS reduced tunnel defects by approximately 97.85% on the RS and 97.82% on the AS. (iii) DR after preheating both sides reduced tunnel sizes by 88.92% on the RS, which is larger than the reduction observed with preheating both sides without DR. Thus, the impact of DR was inconclusive, as the defects were located too far from the surface where DR was applied. Therefore, it can be concluded that the DR process positively influences the reduction of tunnel defects, though its effectiveness is limited by the defect’s proximity to the surface. Nagarajan et al. [50] reported that the distribution of residual stress after undergoing DR with a HG13 ball is confined to a depth of up to 700 μm from the surface. Beyond this depth, the deep rolling process does not significantly influence the surface properties. In contrast, Beghini et al. [51] found that applying a force of 150 N resulted in compressive residual stress extending only 600 μm from the surface. Therefore, if a tunnel defect is located within a critical area, the DR process becomes significant for defect reduction.

**Figure 17:**
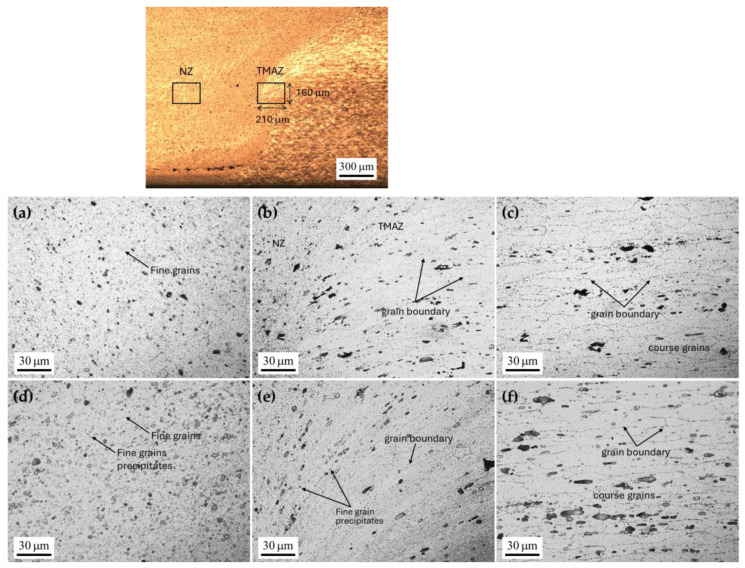
Microstructure zones under non-preheated conditions: (**a**) nugget zone, (**b**) thermo-mechanically affected zone, and (**c**) base zone. Microstructure zones with preheating on both sides: (**d**) nugget zone, (**e**) thermo-mechanically affected zone, and (**f**) base zone.

Figure 17 displays the microstructures of the nugget zone (NZ), base zone (BZ), and thermo-mechanically affected zone (TMAZ). The non-preheated condition, depicted in Figure 17a–c, serves as a baseline for comparison with other conditions. Figure 17a shows fine grains resulting from plastic deformation during welding. Preheating has a more pronounced effect on the NZ compared to other zones, as evidenced by the new fine grains precipitating in the NZ under preheated conditions (Figure 17d), along with a significant reduction in grain size owing to the application of preheating assistance during welding [13] as well as the pressure from the deep rolling process. The recrystallized grain structure in the NZ enhances the strength and toughness of the weld joint, with this contribution being proportional to the grain size in the NZ. In the TMAZ, fine grains are precipitated under preheated conditions (Figure 17e), resulting in an increase in grain boundaries within this zone. In the BZ of the substrate, the predominant grain structure is coarse. However, this zone remains unaffected by the preheating process, regardless of whether preheating plate assistance is applied.

## 4. Conclusions

The effects of a backplate preheating assistance system and the deep rolling (DR) process on the friction stir welding (FSW) of AA6061 joints were investigated with respect to axial force, residual stress, and tunnel defects. The study led to the following conclusions:

- Preheating at 100 °C, 150 °C, 200 °C, and 250 °C reduced the axial force required in the non-preheating condition by 7.33%, 5.84%, 30.24%, and 39.67%, respectively. Based on the observed appearance, force requirements, and achieved temperatures, a preheating temperature of 200 °C was identified as optimal.
- The back plate preheating assistance system provides a more substantial heat input, enhancing material flow and contributing to a reduction in tunnel defects. This system also facilitates deeper penetration of the tool pin end, particularly when preheating is applied to both sides.
- Preheating on the advancing side (AS) resulted in the smallest tunnel defect in the cross-sectional microstructure compared to preheating on the retreating side (RS) or both sides. Specifically, this preheating condition reduced tunnel defect size by 80.15% on the RS and 96.91% on the AS.
- A significant percentage difference in residual stress was observed at the right shoulder under the preheated AS condition, where residual stress changed from 1 MPa to −105 MPa after applying deep rolling pressure. The most compressive residual stress of −137 MPa was observed at the right shoulder under preheating on the AS; however, the effect of preheating was found to be negligible.
- The application of DR post-preheating positively impacted the reduction of tunnel defects. However, its effectiveness was limited by the proximity of the defect to the surface. DR post-preheating on the AS produced the most significant reduction in tunnel defects, decreasing them by approximately 97.85% on the RS and 97.82% on the AS. This improvement exceeded that achieved through preheating alone by 17.7% on the RS and 0.91% on the AS.
- Preheating had a significant effect on the nugget zone, leading to the formation of finer grains and a notable reduction in grain size in the nugget zone under preheated conditions.
- This study highlights the significant effects of preheating assistance, providing insights into the system’s capabilities and configurations to guide future applications and developments. Consequently, preheating techniques can be adapted for various industries by adjusting the heat source to meet specific operational needs. Additionally, investigating dissimilar materials with the preheating system is valuable for exploring optimal conditions and understanding the effects of preheating on different materials.

## Figures and Tables

**Table 1:** Chemical composition of aluminum alloy 6061 in wt.%.

Element	Mg	Fe	Si	Zn	Cr	Ti	Cu	Al
6061	0.867	0.778	0.746	0.241	0.094	0.035	0.062	Bal.

**Table 2:** Fixed parameters of the FSW process and thermal camera setting.

Parameter	Unit	Value
Welding speed	mm/min	65
Plunge depth	mm	2.75
rotational speed	rpm	980
Tool type	-	cylindrical threaded tool
Temperature collecting time	min.	3
Camera resolution	pixels	640 × 480
Frame rate	Hz	30

**Table 3:** Fixed parameters of the deep rolling process.

Parameter	Unit	Value
Ball type	-	HG6
Ball diameter	mm	6
Feed rate	rpm	1400
Pressure	bar	150
Oil flow rate	liters/min.	2

**Table 4:** Preheating and deep rolling conditions.

No.	Preheating		Deep Rolling
	Advancing Side	Retreating Side	
1	−	−	−
2	+	−	−
3	−	+	−
4	+	+	−
5	+	+	+
6	+	−	+
7	−	+	+

+ indicates performing preheat or deep roll, while − indicates not performing preheat or deep roll.

**Table 5:** Appearance of weld formation at different preheating temperatures.

Condition	Appearance
Non-preheating	* 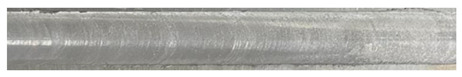 *
100 °C	* 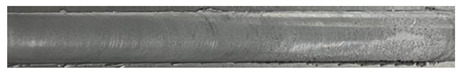 *
150 °C	* 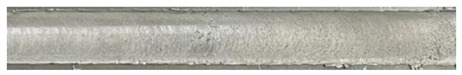 *
200 °C	* 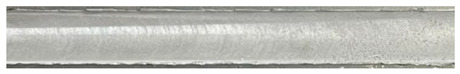 *
250 °C	* 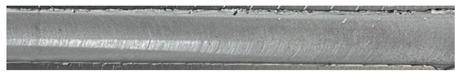 *

**Table 6:** Percentage of difference between residual stress of non-deep rolled and deep rolled.

Preheated Side		Residual Stress (MPa)		
	Deep Rolling	Position 1 (−6 mm)	Position 2 (Center)	Position 3 (+6 mm)
advancing side (AS)	No	−45 ± 6	10 ± 1	1 ± 2
	Yes	−124 ± 2	−56 ± 2	−105 ± 2
	% difference	175.56%	660.00%	10,600.00%
retreating side (RS)	No	−60 ± 5	4 ± 3	−8 ± 5
	Yes	−102 ± 2	−61 ± 2	−137 ± 2
	% difference	70.00%	1625.00%	1612.50%
both sides	No	−23 ± 7	13 ± 2	−45 ± 5
	Yes	−106 ± 2	−62 ± 1	−97 ± 2
	% difference	360.87%	576.92%	115.56%

Note: % difference = (|σ_x, (DR)_ − σ_x, (NDR)_| × 100)/σ_x, (NDR)_; σ_x, (DR)_ = represents the residual stress under deep rolling conditions, while σ_x, (NDR)_ denotes the residual stress without deep rolling.

**Table 7:** Cross-sectional microstructure of stir zone on retreating side and advancing side.

Retreating Side (RS)	Advancing Side (AS)	Observation
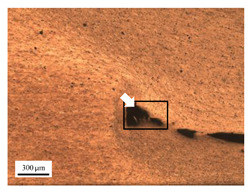 (non-preheated)	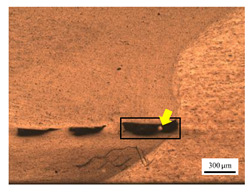 (non-preheated)	**(1) Non-preheating:** Significant tunnel defects were observed across the width of the pin tool at the bottom of the stir zone. These defects are attributed to insufficient material flow and inadequate heat generation, consistent with findings from previous studies [44,45]. The largest tunnel defect on the RS measured 0.0325 mm², as indicated by the white arrow, while the most significant defect on the AS measured 0.0551 mm², as marked by the yellow arrow.
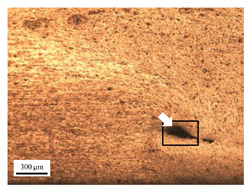 (preheated)	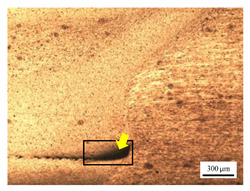 (non-preheated)	**(2) Preheating on RS:** the material flow on the RS is more pronounced compared to the as-welded specimen, likely due to the increased heat input, resulting in a reduced tunnel defect size of 0.0158 mm². On the AS, a tunnel defect measuring 0.0281 mm² is attributed to insufficient heat input. Additionally, the distance between the bottom surface and the tool end on the AS is reduced.
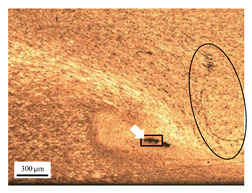 (non-preheated)	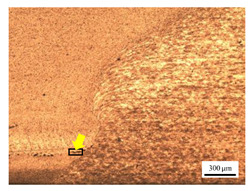 (preheated)	**(3) Preheating on AS:** a grain bond was identified within the circular symbol, accompanied by a tunnel on the RS and several smaller tunnels on the AS. The tunnel on the RS measured 0.0065 mm², as pointed by the white arrow. While on the AS, the defects measured 0.0017 mm², as pointed by the yellow arrow. The increased heat input on the AS facilitated enhanced metal flow, surpassing the effects of preheating on the RS.
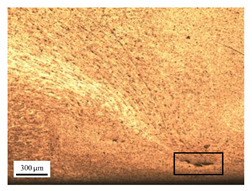 (preheated)	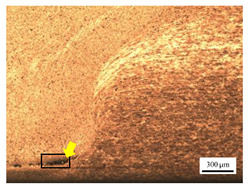 (preheated)	**(4) Preheating both sides:** the tunnel defect on the AS measured 0.0046 mm². On the RS, the tunnel size was 0.0036 mm². The tool end penetrated deepest compared to the preheating conditions on the AS (2) or the RS (3), and it appears symmetric on both the left and right sides. The material flow is well-distributed on both the RS and AS, with narrow holes detected below the tool outline.
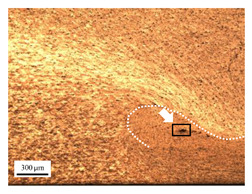 (preheated)	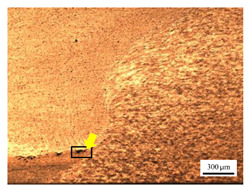 (non-preheated)	**(5) Preheating RS with DR:** compared to condition (2), the holes are smaller and closer together due to the effects of the deep rolling process. The tunnel defect on the RS measured 0.0014 mm², as indicated by the white arrow, while the size on the AS was 0.0018 mm², as indicated by the yellow arrow. However, a cold lap defect was observed on the RS (indicated by the white dashed line), attributed to insufficient flow and mixing of the plasticized metal [46].
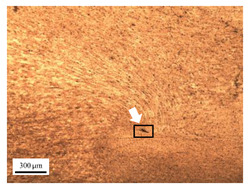 (non-preheated)	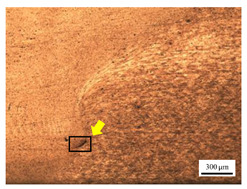 (preheated)	**(6) Preheating AS with DR:** small tunnels were detected only at the corners of the tool outline (indicated by red arrows). No cracks or other grooves were observed. The metal contact at the bottom part is nearly homogeneous. The tunnel defect on the RS (indicated by the white arrow) measured 0.0007 mm², while the size on the AS (indicated by the yellow arrow) was 0.0012 mm².
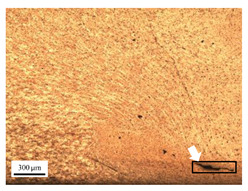 (preheated)	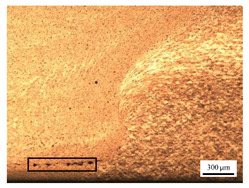 (preheated)	**(7) Preheating both sides with DR:** a narrow tunnel was observed at the bottom of the nugget zone on the RS. Several small tunnels were identified at the interface between the tool outline and the underlying metal. The size of the tunnel defects remained unchanged by the application of deep rolling. The tunnel defect on the RS (indicated by the white arrow) measured 0.0045 mm², but on the AS, various small holes were observed.

## Data Availability

Data used in this study are available upon reasonable request to the corresponding author.
